# COVID-19 and Biomedical Experts: When Epistemic Authority is (Probably) Not Enough

**DOI:** 10.1007/s11673-021-10157-5

**Published:** 2022-01-17

**Authors:** Pietro Pietrini, Andrea Lavazza, Mirko Farina

**Affiliations:** 1 Scuola Alti Studi Lucca, Piazza S. Francesco, 19, 55100 Lucca, LU Italy; 2 Centro Universitario Internazionale, Via Antonio Garbasso 42, 52100 Arezzo, AR Italy; 3 Institute for Humanities and Social Sciences, Universitetskaya St, 1, Innopolis, Republic of Tatarstan Russian Federation 420500

**Keywords:** Public health, Science, Expertise, Ethics

## Abstract

This critical essay evaluates the potential integration of distinct kinds of expertise in policymaking, especially during situations of critical emergencies, such as the COVID-19 pandemic. This article relies on two case studies: (i) herd immunity (UK) and (ii) restricted access to ventilators for disabled people (USA). These case studies are discussed as examples of experts’ recommendations that have not been widely accepted, though they were made within the boundaries of expert epistemic authority. While the fundamental contribution of biomedical experts in devising public health policies during the COVID-19 pandemic is fully recognized, this paper intends to discuss potential issues and limitations that may arise when adopting a strict expert-based approach. By drawing attention to the interests of minorities (disenfranchized and underrepresented groups), the paper also claims a broader notion of “relevant expertise.” This critical essay thus calls for the necessity of wider inclusiveness and representativeness in the process underlying public health policymaking.

## Introduction

When severe emergency health situations arise, biomedical and public health experts are consulted and their recommendations are closely followed. During the current COVID-19 pandemic, experts have been called upon to make particularly relevant decisions for the whole society, to an extent that likely had never been experienced before. In many cases, leaders and governments have faithfully, and sometimes almost uncritically, trusted biomedical expertise in order to formulate political decisions and implement public health policies aimed at protecting the well-being of society. This can be explained by both the trust and confidence that our culture has bestowed upon experts and the attempt of political leaders and governments to overturn, at least in part, the responsibility for the required restrictive measures, so to defuse the public reaction.

In this paper, we examine the role that experts played in guiding public health debates during the COVID-19 pandemic. Because of its enormous impact on the whole society, we argue that such a role should be subject to closer scrutiny.

In section 2, we reflect on what an expert exactly is. In section 3, we look at the role that biomedical experts have played in the current COVID-19 pandemic. In section 4, we analyse the moral, ethical, and political implications of recommendations by biomedical experts, focusing on two case studies (herd immunity and priority rules). In section 5, we note a crucial lack of inclusiveness and representativeness in the decisions implemented by politicians based on the mere implementation of recommendations made by biomedical experts. In section 6, we conclude by indicating the need for a more participated, less fragmented, decisional process in policymaking.

## Experts as Ultimate Decision-Makers

The philosopher Alvin Goldman has been trying to define what exactly an expert is for most of the last twenty years. For Goldman (2001) “an expert in domain D is someone who possesses an extensive fund of knowledge (true belief) and a set of skills or methods for apt and successful deployment of this knowledge to new questions in the domain” (92). According to Goldman then, the main function of an expert is sharing some knowledge for the benefit of someone else. Goldman’s account of expertise, albeit very influential, is not the only one developed in the literature.

As a matter of fact, further elaborating on Goldman’s definition, Elizabeth Fricker proposed a very influential notion of expertise:

S is an expert about P relative to H at t just if at t, S is epistemically well enough placed with respect to P so that were she to have, or make a judgment to form a conscious belief regarding whether P, her belief would almost certainly be knowledge; and she is better epistemically placed than H to determine whether P. (2006, 233)

Thus, an expert, at a minimum, is an individual who is epistemically placed with respect to a proposition P in such a way that any beliefs she will form about that proposition P will more likely than not be true. In other words, “experts possess an improved epistemic stance (or greater epistemic authority) over non-experts and, by virtue of this, they can make informed decisions and accurate predictions that can increase the welfare of their communities” (Lavazza and Farina 2020, “The state of affairs,” ¶2).

Thus, by definition, experts tend to be experts in their specific domain of expertise. This means that they tend to look at problems from their epistemic perspective—often ignoring alternative ones—which may result in a partial view. To give a few examples: virologists focus on the mechanisms of viral replication; epidemiologists look at the data about the spread of the contagion; anaesthetists are concerned with the clinical management of intensive care units (ICUs). Although these different biomedical experts may collaborate and work together on a clinical ground, their disciplinary focus and training, in their everyday life, still determines a rather reductive approach to complex issues.

If disciplinary focus, on the one hand, has been fundamental for relevant advancements in medical care, on the other hand, it could also lead to the fragmentation of the decisional process. We believe that if this reductive approach often adopted by biomedical experts is not framed within forms of a holistic, collaborative, and participative thinking, it may encounter the risk of becoming a form of scientism (the idea that there is no truth to be found in any other area except in the sciences) and consequently of being unrepresentative. Ultimately, a strictly disciplinary and narrow approach may ignore issues that are crucial for the overall well-being of large swathes of the population, especially for the disadvantaged and disenfranchized groups.

So, why do we trust experts? There are several reasons for this. First, experts usually provide successful indications. Think about how life conditions and average life expectancy have been progressively improving over the last one hundred years. Thanks to the progress in science and medicine, we have escaped what Hobbes called the “solitary, poor, nasty, and brutish” nature of humanity (Hobbes, 1651/2008 ch.13). Second, resorting to expertise helps to reduce controversy. The expert is generally seen as a truth carrier, who can illuminate problematic aspects of our existence, through rationality and objectivity.

## The Specificity of the COVID-19 Crisis

In recent years, however, there has been an open tendency to distrust experts and their knowledge (notable case studies involve climate change and Brexit), not only among laypeople but also among political circles (the former American President Donald Trump, the British Prime Minister Boris Johnson and the Brazilian President Jair Bolsonaro are probably the most paradigmatic examples of such a distrust).

Yet, during the COVID-19 crisis, biomedical experts have been called upon to manage the emergency, even in countries in which they previously had been harshly contested, like the United Kingdom (Clarke and Newman 2017). This likely happened because both policymakers and citizens experienced first-hand the dangerousness of the virus and the costs of an ineffective response.

However, there was no general agreement among biomedical experts. This is a common situation in science, albeit often overlooked (Coady 2006). Many experts suggested technical recommendations for the containment of the infection, including a variety of non-pharmacological interventions, such as the lockdown. Others advised against the implementation of such measures for epistemic, constitutional, and economic reasons, and, for example, suggested to pursue herd immunity, as in United Kingdom or Sweden (Lavazza and Farina 2020, 2021a, 2021b; Farina and Lavazza 2021a, b).

In the United Kingdom, a few experts, including the U.K.’s chief scientist adviser, provided scientific arguments to support the government’s initial scepticism about the possibility of the contagion taking place on a large scale. Top advisers initially endorsed a prudent strategy to fight the spreading of the coronavirus, based on the Contain-Delay-Mitigate-Research: as a result, those who had symptoms were not tested, contrary to WHO’s recommendations, and the state did not enforce either quarantine or isolation within the general population.

It was only after the release of a report predicting 500,000 deaths in the United Kingdom if the pandemic was not properly tackled (Ferguson et al. 2020), that the government decided to change its strategy and announced more drastic measures—including school closures throughout the country and restrictions on the people’s freedom of movement and assembly—to prevent the contagion from spreading further. The official justification for this change in policy was that scientific data had changed. However, the justification provided was quickly refuted as scientifically unsound by leading scholars (such as Horton 2020).

As it emerged in the following months, achieving herd immunity in the case of a virus like the Sars-CoV-2 is essentially impossible. This because, as Brett and Rohany (2020, 25897) demonstrated:

1. social distancing must initially reduce the transmission rate to within a narrow range; [and] 2. to compensate for susceptible depletion, the extent of social distancing must be adaptive over time in a precise yet unfeasible way; and 3. social distancing must be maintained for an extended period to ensure that the healthcare system is not overwhelmed.

For these reasons, it is reasonable to assume that a wider panel of experts, more attentive to the social and psychological consequences of the embraced strategy, could have led to the adoption of decisions, such as an earlier lockdown, that could have considerably lowered the death toll (an instructive comparison can be drawn with Ireland, see Colfer 2020).

In the anticipation of the arrival of vaccines, herd immunity may have seemed a reasonable strategy, mostly based—according to the U.K. government—on the risk of a “behavioural fatigue,” which could have possibly undermined “the effectiveness of the lockdown, as people would start violating the recommendation to stay home” (Sibony 2020, 354). However, most behavioural scientists immediately dismissed such a claim as scientifically ungrounded (Sibony 2020).

With respect to vaccines, it should be noted that, at the time of writing, it remains yet to be determined to what extent they prevent the transmission of the virus, as it appears that vaccinated individuals may still carry the virus and infect vaccinated and unvaccinated subjects (even though at a lower rate). In addition, vaccine roll-out is dramatically uneven (Farina and Lavazza 2021a); the emergence of new variants is likely to modify any predictions about herd immunity; furthermore, immunity may not last forever; and, no less important, vaccination may induce an unjustified sense of safety and lead people to adopt more relaxed behaviours, abandoning those preventive measures that should still be maintained until the virus has been completely eradicated (Aschwanden 2021).

Another example of disagreement among biomedical experts concerns the adoption of criteria for accessing life-saving ventilators in some U.S. states, when the available devices became fewer than the patients needing them. These criteria incorporated specific priorities (e.g., the exclusion of mentally disabled individuals or people with specific pathologies), which were deemed by some experts to be the most effective or the most suitable for the emergency situations (Baker and Fink 2020).

Specifically, in March 2020, Alabama issued a state policy according to which people with “severe or profound mental retardation” and “moderate to severe dementia” were “unlikely candidates” for receiving a life-saving medical device. After complaints from organizations of disabled people, the state withdrew the policy and introduced new and more generic guidelines, which did not discriminate on the basis of cognitive status.

On a similar vein, the state of Washington recommended that patients with “loss of reserves in energy, physical ability, cognition and general health” be reserved for palliative care. These guidelines aroused the protests of several organizations for the defence of disabled people, which appealed to the federal Government to impose on local authorities and hospitals the principle that disabled people ought to be entitled to the same treatment as all the other COVID-19 patients, on the grounds that the above-mentioned exclusion criteria were utterly unfair and discriminatory (Disability Rights Education & Defense Fund 202).

In response, the Office for Civil Rights at the U.S. Department of Health and Human Services stated that, based on Section 1557 of the Affordable Care Act and Section 504 of the Rehabilitation Act, “persons with disabilities should not be denied medical care on the basis of stereotypes, assessments of quality of life, or judgments about a person’s relative ‘worth’ based on the presence or absence of disabilities or age” (OCR 2020, 1).

A systematic literature review conducted in the United States at the beginning of 2020 revealed that twenty-six states had publicly available ventilator guidelines (Piscitello et al. 2020), eleven of which recommended certain exclusion criteria in adults. Specifically, eight states envisaged exclusion criteria for “irreversible severe neurologic injury or disease” and three states had such criteria applied for “severe dementia.” The protests that the formulation of these guidelines triggered, both among politicians and intellectuals, mostly regarded the bioethical aspects related to their adoption (Bledsoe et al. 2020; Andrews et al. 2020; McGuire et al. 2020; Chen and McNamara 2020; cf. Lavazza and Farina 2020). Overall, this widespread reaction indicates that the decision of adding exclusion criteria for mental disabilities likely was made following mere biomedical priorities in the absence of ethical experts and/or representatives from disadvantaged and disenfranchised groups.

It is worth noting that roughly in the same period, other experts proposed different frameworks to allocate ventilators in cases of shortages. In particular, White and Lo (2020, E1) argued that those criteria of exclusion (namely, severe cognitive impairments) “are not explicitly justified and they are ethically flawed” because they “are selectively applied to only some types of patients.” In addition, “such exclusions violate a fundamental principle of public health ethics: use the means that are the least restrictive to individual liberty to accomplish the public health goal.”

## Biomedical Experts and Political Decisions

The two cases we discussed above—herd immunity and priority rules—are examples of decisions that have been contested not necessarily on a technical (epistemic) level but rather in terms of ethical principles and axiological values. In the case of herd immunity, several researchers both in the United Kingdom (Horton 2020b) and in Sweden (Robertson 2020) have attacked the public policies implemented by politicians for failing to fully acknowledge the role of asymptomatic carriers and highlighted the potential catastrophic impact of this decision on large swathes of population (Horton 2020b), especially among the weakest components of society (Chowkwanyun et al. 2020), including the elderly and the minorities (underrepresented and/or disenfranchized groups: Yancy 2020). With respect to priority rules, the criteria adopted for accessing life-saving ventilators have been strongly criticized, as we already discussed, as discriminatory and were modified or withdrawn because of their incompatibility with federal legislation (Novak 2020).

The guidelines from individual states may reflect the positions of health experts alone or the political orientations of legislators as well. However, proposals or decisions made by experts—based on their technical expertise and presumably in good faith, i.e., without explicit cultural, ideological, political, or religious views or biases being at play—cannot be justified simply by their epistemic authority. The potential adoption of the (cognitive) disability criterion to rank (mentally) disabled individuals at the bottom of the list of those allowed to access intensive care, as shown by the reactions provoked in the United States, must be publicly justified with reference to the specific reasons that support the general interests of society (Lavazza and Farina 2021a).

These cases clearly show that recommendations and decisions offered by biomedical experts, while being epistemically sound, do also have important consequences at moral, ethical, and “political” levels. Biomedical experts know with no doubt far more than the average citizens about viruses and epidemics; however, in the cases discussed above, their epistemic authority was not, *per se*, sufficient to ensure that their recommendations were such to produce broadly shared, uncontroversial health policies. Most importantly, it became evident that those recommendations failed to consider and properly value the needs of the society a whole (Lavazza and Farina 2021b).

Therefore, we believe that in such complex situations in which the health and safety of the whole society is at stake, the decision-making process at a political level ought to be not merely guided by biomedical expert’s opinions, mostly because they can be either diametrically divergent and/or have questionable ethical implications, as in the cases of herd immunity and of the access to life-saving ventilators discussed above. Rather, political authorities should promote and articulate their discussions within the wider society (Rawls, 1993), with attention to ethical and moral principles as well as to constitutional rights (Jasanoff 2012).

In other words, we firmly believe that our society cannot and should not do without science (Haack 2016), yet we also think that science at times may be, like all human enterprises, fallible, imperfect, and susceptible to corruption (Haack 2011). We thus believe that we should avoid the risk of scientism, especially at times of emergency, which we deem would be no less dangerous for our culture and for the proper functioning of our democracies than the anti-scientific cynicism that science aims to fight.

## The Challenge of Inclusiveness

Scientism essentially asserts that all our knowledge is based on natural phenomena and should be reduced to the study of their properties and relations. This position, in recent years, has been advocated by many prominent intellectuals both in philosophy and in the natural sciences (e.g. Ross et al. 2007).

However, we believe that experts relying just on such a strong naturalistic approach (the thought that science is the only method for obtaining reliable knowledge) when called upon to advise decision-makers or formulate and design public health policies, should necessarily consider aspects, values, and virtues cherished also by other people. Such values may not necessarily be anti-scientific and yet be complementary to those. Elsewhere, two of the authors propose the idea of experts in action to substantiate this intuition (Lavazza and Farina 2021b, 145). Experts in action are “individuals with above average knowledge, certified and achieved in the most objective and repeatable possible way, in a specific field, who use their skills and methods to translate this knowledge into decisions, actions or suggestions for decisions (or actions) that concerns a community larger than that of the experts themselves.”

The two case studies we described above can be re-interpreted, we believe, within this new framework. These case studies highlight the need to consider the ethical, social, economic, and political features underlying the COVID-19 pandemic (Farina and Lavazza 2020). Specifically, when it is necessary to make decisions that involve predictions about people’s behaviour, values, and symbolic elements, simply resorting to epistemic authority cannot be enough because these are phenomena that do not have a precise quantitative and objective characterization.

In this sense, the idea of inclusiveness of science does not mean that any epistemic agent can provide a contribution in terms of knowledge and decision-making competence on these aspects. Even in ethics (where the disagreement persists and cannot be resolved simply by performing experiments) and in social sciences one can distinguish between experts who are professionally trained and are respected scholars in their discipline and individuals who rely only on common sense and their own subjective experience. However, inclusiveness also means involving representatives of points of view, interests, and groups for which it is understood that they are not adequately represented.

This is the way in which scientific rigor ought to serve inclusiveness and diversity; that is,

… the ways in which people differ, encompassing all the characteristics that make one individual or group distinctive. The dimensions of diversity include but are not limited to: (i) ethnic or national origins, skin colour or nationality, (ii) gender, gender identity and gender expression, (iii) sexual orientation, (iv) background (socio-economic status, immigration status or class), (v) religion or belief (including absence of belief), (vi) civil or marital status, (vii) pregnancy and maternity, paternity, parental leave, (viii) age and (ix) disability. (Urbina-Blanco et al. 2020, 18307).

Science, we argue, should be fair in treating and granting access and opportunity that lead to the advancement of all people. Accordingly, inclusion should be understood as **“**the act of creating an environment in which any individual or group feels (i) welcomed, (ii) safe, (iii) supported, (iv) respected and (v) valued to participate” (Ibidem).

In a crisis such as that of COVID-19, in which two distinct, though intertwined, aspects—the lives of hundreds of thousands of people and the economic conditions of millions—are at stake and may present conflicting features, we do need biomedical experts. However, we ought to go beyond mere biomedical competences, so that public health policies be grounded not only on their epistemic authority. If we are to protect the overall welfare of society, including the well-being of its most disadvantaged and disenfranchized groups, public health policies should embrace a wider conception of “relevant expertise” (Williams 1998).

Recent studies have shown that, in the United States, African-Americans are dying of COVID-19 at three times the rate of white people (Khunti et al. 2020; Kirby 2020). The question arises whether public health policies devised by a wider range of experts within a better integrated decisional process could have resulted in a more effective protection of these groups. An additional question follows about whether, in emergency situations, we should give voice to experts whose expertise is not based solely on “epistemic status” but rather on experience or political advocacy, of the homeless, the immigrant, or other disenfranchized groups.

Let us consider the following example. In August 2020, the WHO Regional Office for Europe has set up a Pan-European Commission on Health and Sustainable Development to rethink policy priorities in light of the COVID-19 pandemic.

After identifying and reviewing the relevant evidence, the Commission will draw lessons from the ways in which the health systems in different countries have responded to the COVID-19 pandemic and will make recommendations on investments and reforms to improve the resilience of health and social care systems. The Commission will also seek to build consensus on these recommendations and to elevate health and social care as societal and political priorities, recognized as being critical to both sustainable development and social cohesion. (World Health Organization 2020)

The Commission was chaired by Professor Mario Monti, former Italy’s Prime Minister and a former European Commissioner. It consisted of former heads of states and governments, distinguished life scientists and economists, heads of health and social care institutions, and leaders of the business community and financial institutions. The idea underlying the establishment of this Pan-European Commission was to have a variety of experts with different skills and former political representatives serving on a committee, so to guarantee the formulation of more balanced and realistic public health policies. The Commission’s work culminated in a report with recommendations on investments and reforms to improve health and social care systems. We praise this attempt at inclusion, which is certainly significantly better than the approach at the onset of the pandemic (when—in many countries—exclusion of potentially relevant non-medical expertise was rather the norm, likely triggered by the panic generated by the pandemic); however, we cannot help but noting that in the Pan-European Commission there were no representatives of civil society, NGOs, minorities and less advantaged groups, as well as of religions.

Based on the above considerations, we argue that a more inclusive conception of science is one in which experts in action make decisions based not just on epistemic soundness or efficiency but also consider the needs of communities that are larger than that of the experts themselves. These experts, in our view, should therefore be aware of the diversity of interests at stake and consider the possibility of having sometimes a non-univocal and objective evaluation of complex situations (Mouter et al. 2020). This, in our view, does not mean devaluing science or reducing its role in society but rather quite the opposite. This seems to call for a more inclusive conception of science and a more liberal understanding of experts.

## Conclusion

We believe that in times of emergency, we should be drawing on every type of potentially relevant expertise across the humanities, social and natural sciences, and on insights from the wider society as well. In other words, we should avoid the risk of any potential form of scientism, intended as closure of science to any other forms of knowledge and normativity sources. Science, as Toulmin once claimed (Toulmin 1972), cannot arrogate to itself the exclusive authority on issue that impact the whole of humanity and that often require global (multi-parties) self-reflection. This is because “that process of global self-reflection must begin with greater humility on the part of science, coupled with deeper awareness of questions that science cannot even properly pose, let alone claim the right or capacity to answer on its own” (Jasanoff et al. 2019, 270).

As we have shown in this paper, science remains fundamental (indeed, it was a supplement of scientific reflection that in the end led Britain to abandon herd immunity). However, when scientific decisions impact directly on the lives of millions of people, science ought to assume an attitude of epistemic openness, without giving up its method and its ability to be effective and efficient.

For these reasons, echoing the philosopher and legal scholar Melissa Williams, we claim that “a fair and just public discourse needs at least some direct representation of the voices of those who are minorities or live in dependence because the majority groups (here experts) do not share their particular history and experience” (Williams 1998, 131 quoted in Schicktanz, Schweda, and Wynne 2012). On these grounds, we therefore call for a more inclusive, more participated, and less fragmented decisional process in the formulation of public health policies, especially at times of emergency.
